# Quelling an innate response to dsRNA

**DOI:** 10.18632/oncotarget.5110

**Published:** 2015-08-06

**Authors:** Kristen M. Ogden, B. V. Venkataram Prasad

**Affiliations:** Verna and Marrs McLean Department of Biochemistry and Molecular Biology, Baylor College of Medicine, Houston, Texas, USA

**Keywords:** Immunology and Microbiology Section, Immune response, Immunity

The interferon inducible oligoadenylate synthetase (OAS)/RNase L pathway is an innate defense against invading viral pathogens that is linked to cell survival [reviewed in 1]. OAS family proteins are pattern recognition receptors that recognize double-stranded RNA (dsRNA), a pathogen-associated molecular pattern. In the presence of dsRNA, which is produced during the replication cycle of a broad array of viruses, cytoplasmic OAS proteins become activated to synthesize 2′-5′-linked oligoadenylates (2-5A) from ATP. The only clearly established function for 2-5A is to activate latent RNase L, which then cleaves single-stranded RNA on the 3′ sides of UpAp and UpUp dinucleotides. This RNase L activity can inhibit viral replication directly, by cleaving the genome of single-stranded RNA viruses or the mRNA of any virus, or indirectly, by impairing host machinery required for viral replication through cleavage of cellular mRNA and rRNA. Additionally, the RNA products of RNase L cleavage can upregulate interferon expression, via signaling through RIG-I-like receptors, and stimulate NLRP3 inflammasome activation.

Mounting evidence underscores the importance of the OAS/RNase L response in host defense against viral infection. OAS and/or RNase L have been shown to inhibit the replication of viruses with both RNA and DNA genomes, including West Nile virus, chikungunya virus, respiratory syncytial virus, dengue virus, hepatitis C virus, and herpes simplex virus-1. Perhaps not surprisingly, viruses from diverse families have evolved a variety of mechanisms to antagonize the OAS/RNase L pathway [1]. One such strategy, which is shared by group A and B rotaviruses (dsRNA genome) and group 2a betacoronaviruses (ssRNA genome), is to encode a 2H phosphodiesterase that directly cleaves 2-5A [2–3].

While the OAS/RNase L response helps protect cells from viral infection, it also can inhibit cell proliferation and induce apoptosis. Thus, this pathway is tightly regulated, primarily through modulation of the potent chemical messenger, 2-5A. Cellular proteins that degrade 2-5A and may act as negative regulators of the OAS/RNase L pathway include A kinase anchor protein 7 (AKAP7), phosphodiesterase 12 (PDE12), and ectonucleotide pyrophosphatase/phosphodiesterase 1 (ENPP1). These proteins, which differ in structure, protein family, and subcellular localization, may function at distinct sites to rapidly remove 2-5A and restore homeostasis following OAS activation.

Recently published structures of a viral 2H phosphodiesterase that cleaves 2-5A, the C-terminal domain (CTD) of the group A rotavirus VP3 protein, in the absence and presence of 2-5A [3-4] shed light on the ligand-binding and catalytic mechanism of these enzymes and on the likely evolutionary origins of viral 2′,5′-phosphodiesterases. Both the rotavirus VP3 CTD and cellular AKAP7 are members of the 2H phosphoesterase superfamily that cluster phylogenetically with one another and with the coronavirus ns2 protein [5]. Purified VP3 CTD forms a seven-stranded, concave β-sheet, at the base of which reside the two HφTφ catalytic motifs (φ = hydrophoic) that typify 2H superfamily phosphoesterases, and two flanking α-helical regions. This structure is strikingly similar (RMSD 3.0Å) to that of the AKAP7 central domain [6]. However, the viral 2H phosphoesterase is more compact, lacking two α-helical regions and two β-strands that are present in the cellular enzyme. Several of the residues in the catalytic cleft that contact 2-5A in the VP3 CTD/2-5A co-crystal structure align with residues that AKAP7 uses to engage AMP and that are conserved among other 2H phosphoesterases known or predicted to cleave 2-5A [3,6]. Thus, these viral and cellular 2′,5′-phosphodiesterases likely employ a common mechanism of substrate recognition and catalysis. These observations provide support for the hypothesis that an ancestral virus usurped a cellular 2′,5′-phosphodiesterase, such as AKAP7, and streamlined the enzyme to accommodate the compact viral genome. The strong similarities between the rotavirus VP3 CTD and AKAP7, in terms of structure and of substrate recognition and catalytic mechanisms, suggest that small-molecule therapeutics designed to target viral 2′,5′-phosphodiesterases will have to be evaluated carefully for potential effects on cellular enzymes that degrade 2-5A, including AKAP7.

A recent study examining HeLa cells lacking PDE12 and PDE12 inhibitors provides evidence that this enzyme modulates cellular 2-5A levels and suggests a role in cellular resistance to viral infection. HeLa cells lacking PDE12 exhibited increased levels of 2-5A in response to dsRNA [7]. Viral spread, but not early viral replication, was delayed in these cells. Treatment of wild-type cells with inhibitors that bind PDE12 modulated 2-5A levels, activated RNase L, and conferred some level of resistance to infection by encephalomyocarditis virus, human rhinovirus, and respiratory syncytial virus in wild-type HeLa cells. Co-crystallization of PDE12 with one of the inhibitors revealed that it is bound in the putative 2-5A binding site. These findings suggest that inhibitors of PDE12 and perhaps of other enzymes that modulate 2-5A in the cell may provide a new avenue for broad-spectrum antiviral drug development. However, in light of the antiproliferative and apoptotic potential of the OAS/RNase L pathway, it will be important to monitor effects of such inhibitory compounds on cell viability and to determine whether they bind other cellular and viral 2-5A-cleaving enzymes or RNase L.
